# Diel Variation in Gene Expression of the CO_2_-Concentrating Mechanism during a Harmful Cyanobacterial Bloom

**DOI:** 10.3389/fmicb.2016.00551

**Published:** 2016-04-22

**Authors:** Giovanni Sandrini, Robert P. Tann, J. Merijn Schuurmans, Sebastiaan A. M. van Beusekom, Hans C. P. Matthijs, Jef Huisman

**Affiliations:** ^1^Department of Aquatic Microbiology, Institute for Biodiversity and Ecosystem Dynamics, University of AmsterdamAmsterdam, Netherlands; ^2^Department of Aquatic Ecology, Netherlands Institute of EcologyWageningen, Netherlands

**Keywords:** carbon dioxide, climate change, CO_2_-concentrating mechanism, gene expression, harmful algal blooms, lakes, *Microcystis aeruginosa*

## Abstract

Dense phytoplankton blooms in eutrophic waters often experience large daily fluctuations in environmental conditions. We investigated how this diel variation affects *in situ* gene expression of the CO_2_-concentrating mechanism (CCM) and other selected genes of the harmful cyanobacterium *Microcystis aeruginosa*. Photosynthetic activity of the cyanobacterial bloom depleted the dissolved CO_2_ concentration, raised pH to 10, and caused large diel fluctuations in the bicarbonate and O_2_ concentration. The *Microcystis* population consisted of three C_i_ uptake genotypes that differed in the presence of the low-affinity and high-affinity bicarbonate uptake genes *bicA* and *sbtA*. Expression of the bicarbonate uptake genes *bicA*, *sbtA*, and *cmpA* (encoding a subunit of the high-affinity bicarbonate uptake system BCT1), the CCM transcriptional regulator gene *ccmR* and the photoprotection gene *flv4* increased at first daylight and was negatively correlated with the bicarbonate concentration. In contrast, genes of the two CO_2_ uptake systems were constitutively expressed, whereas expression of the RuBisCO chaperone gene *rbcX*, the carboxysome gene *ccmM*, and the photoprotection gene *isiA* was highest at night and down-regulated during daytime. In total, our results show that the harmful cyanobacterium *Microcystis* is very responsive to the large diel variations in carbon and light availability often encountered in dense cyanobacterial blooms.

## Introduction

Harmful cyanobacterial blooms are a recurring problem in many eutrophic lakes worldwide (Chorus and Bartram, 1999; Huisman et al., 2005; Michalak et al., 2013). Cyanobacteria can produce a variety of hepatotoxins, gastrointestinal toxins, and neurotoxins causing liver, digestive and neurological disease in birds and mammals, including humans (Carmichael, 2001; Codd et al., 2005). This may lead to the closure of water bodies for recreational use, drinking or irrigation water, and aquaculture (Verspagen et al., 2006; Qin et al., 2010; Steffen et al., 2014). Cyanobacterial blooms are often favored by excessive nutrients and high temperatures (Jöhnk et al., 2008; Kosten et al., 2012), and it is foreseen that rising CO_2_ concentrations and global warming will further promote harmful cyanobacterial blooms (Paerl and Huisman, 2008; O’Neil et al., 2012; Verspagen et al., 2014; Visser et al., 2016).

Yet, effects of daily fluctuations in environmental conditions on harmful cyanobacterial blooms remain poorly investigated. The day–night cycle leads to large changes in the activity of phototrophic organisms. The high photosynthetic activity of cyanobacterial blooms during daytime can deplete the dissolved CO_2_ (CO_2_(aq)) concentration, causing a concomitant increase in pH (Ibelings and Maberly, 1998; Balmer and Downing, 2011; Gu et al., 2011). Conversely, at night, cyanobacteria obtain their energy from respiration of the stored carbon compounds, releasing CO_2_ back in the water column. Cyanobacteria adjust to this daily variation by changes in gene expression and the built-up or break-down of specific enzymes and cellular components (Kucho et al., 2005; Straub et al., 2011; Brauer et al., 2013). In particular, the daily excursions in the availability of both light and inorganic carbon (C_i_) imply that cyanobacteria may not only acclimate their light reactions of photosynthesis, but may also adjust their C_i_ uptake machinery to the diel cycle.

Cyanobacteria have evolved a CO_2_-concentrating mechanism (CCM) to efficiently fix CO_2_ at a wide range of C_i_ conditions (Kaplan and Reinhold, 1999; Price et al., 2008; Raven et al., 2012; Burnap et al., 2015). The cyanobacterial CCM is based on the uptake of dissolved CO_2_ and bicarbonate by different uptake systems and subsequent concentration of C_i_ in specialized compartments, called carboxysomes, that also contain the CO_2_ fixing enzyme RuBisCO. Five different C_i_ uptake systems are known in cyanobacteria, three for bicarbonate uptake and two for CO_2_ uptake (Price, 2011). The bicarbonate transporter BCT1 and the two CO_2_ uptake systems are present in most freshwater cyanobacteria (Badger et al., 2006; Rae et al., 2011; Sandrini et al., 2014). BCT1 is directly ATP-dependent, and combines a high affinity with a low flux rate (Omata et al., 1999). The two redox-dependent CO_2_ uptake systems, NDH-I_3_ and NDH-I_4_, have contrasting properties: NDH-I_3_ has a high affinity and low flux rate, while NDH-I_4_ has a low affinity and high flux rate (Maeda et al., 2002; Price et al., 2002). The presence of the other two bicarbonate uptake systems, BicA and SbtA, varies among freshwater cyanobacteria (Badger et al., 2006; Rae et al., 2011; Sandrini et al., 2014). Both BicA and SbtA are sodium-dependent symporters, where SbtA usually has a high affinity but low flux rate, and BicA has a low affinity but high flux rate (Price et al., 2004; Du et al., 2014). We recently compared CCM gene sequences of 20 strains of the harmful cyanobacterium *Microcystis aeruginosa* (Kützing; Sandrini et al., 2014). Interestingly, some strains lacked the high-flux bicarbonate uptake gene *bicA*, whereas others lacked the high-affinity bicarbonate uptake gene *sbtA*. Hence, in *Microcystis*, three different C_i_ uptake genotypes can be distinguished: *bicA* strains (with *bicA* but no *sbtA*), *sbtA* strains (with *sbtA* but no or incomplete *bicA*), and *bicA* + *sbtA* strains.

In laboratory studies, several cyanobacterial CCM genes were shown to be responsive to changes in both C_i_ availability and light intensity (Hihara et al., 2001; Wang et al., 2004; Schwarz et al., 2011; Straub et al., 2011; Burnap et al., 2013; Sandrini et al., 2015a). However, whereas lab studies usually focus on a single strain, cyanobacterial blooms often consist of multiple strains (Saker et al., 2005; Kardinaal et al., 2007). Recent work shows that different strains may respond differently to changes in CO_2_ levels (Van de Waal et al., 2011; Sandrini et al., 2014, 2015b), which may complicate how daily fluctuations in C_i_ availability affect gene expression and cellular physiology of cyanobacterial communities in lakes. Two lake studies used metatranscriptomics to study diel variation in cyanobacterial gene expression (Penn et al., 2014) or to compare gene expression between different locations (Steffen et al., 2015), yet detailed information on C_i_ concentrations and differences in expression of, for example, the C_i_ uptake genes were missing. Another interesting field study used RT-qPCR to investigate diel variation in the expression of CCM and other selected genes of a *Synechococcus* strain in the hot spring microbial mats from Yellowstone National Park (Jensen et al., 2011). However, detailed data about the C_i_ conditions in the mats were not presented.

In this study, we investigate diel variation in gene expression patterns of *Microcystis* in a dense cyanobacterial bloom of a eutrophic lake. *Microcystis* is a ubiquitous harmful cyanobacterium that often threatens water quality by the production of dense blooms and hepatotoxins called microcystins (Carmichael, 2001; Huisman et al., 2005; Qin et al., 2010; Dittmann et al., 2013). We quantified expression of the CCM genes as well as a few other selected genes of *Microcystis* using RT-qPCR. Furthermore, we monitored diel changes in environmental conditions, to link observed gene expression patterns to fluctuations in C_i_ and light availability. Our study provides a better understanding of the daily variations in the CCM during dense cyanobacterial blooms.

## Results

### Community Composition

**FIGURE 1:**
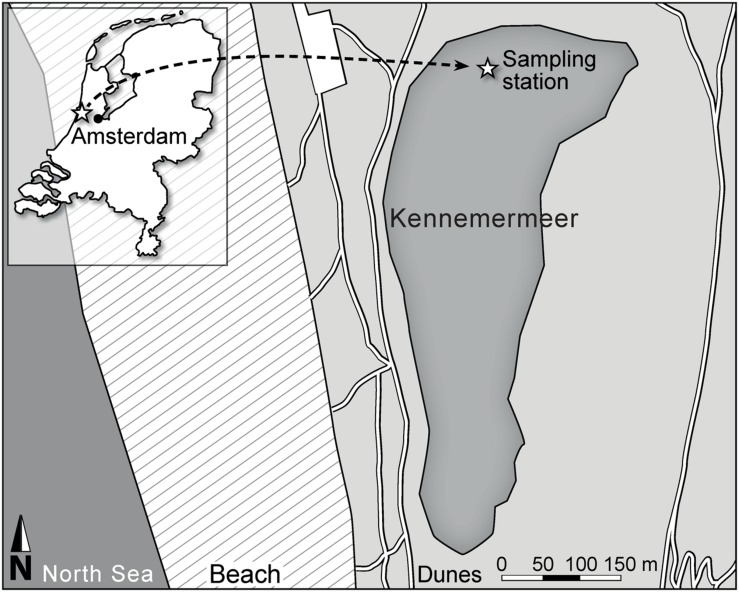
**Map of Lake Kennemermeer with the sampling station**.

Changes in gene expression and environmental conditions were monitored in Lake Kennemermeer (**Figure 1**) for 24 h, from 13:00 to 13:00 the next day on July 17 and 18, 2013. At this time, the lake was dominated by cyanobacteria comprising >97% of the total phytoplankton biovolume. The three most abundant cyanobacterial groups included Pseudanabaenaceae (biovolume: 380 mm^3^ L^-1^; cell counts: 10.9 × 10^9^ cells L^-1^), *Anabaenopsis hungarica* (67 mm^3^ L^-1^; 53 × 10^7^ cells L^-1^) and small Chroococcales (40 mm^3^ L^-1^; 24.6 × 10^9^ cells L^-1^), whereas *Microcystis* spp. represented a smaller portion of the cyanobacterial community (1 mm^3^ L^-1^; 70 × 10^6^ cells L^-1^). We detected two morphotypes of *Microcystis*, known as *M. aeruginosa* and *M. flos-aquae* (Komárek and Anagnostidis, 1999). These two morphotypes differ in morphology of the *Microcystis* colonies, but belong to the same genetic species *M. aeruginosa* (Kützing) according to the Bacterial Code (Otsuka et al., 2001).

Quantification of the C_i_ uptake genotypes of *Microcystis* was based on qPCR using a series of primers targeting the *bicA* and *sbtA* genes in the sampled gDNA. The results revealed that the total *Microcystis* population consisted of *bicA* strains (23 ± 1%), *sbtA* strains (15 ± 1%) and *bicA* + *sbtA* strains (62 ± 2%). Hence, all three C_i_ uptake genotypes of *Microcystis* were present in the lake. Quantification of the microcystin synthetase gene *mcyB* showed that 82 ± 5% of the *Microcystis* population was potentially toxic.

### Diel Variation in Environmental Conditions

**FIGURE 2:**
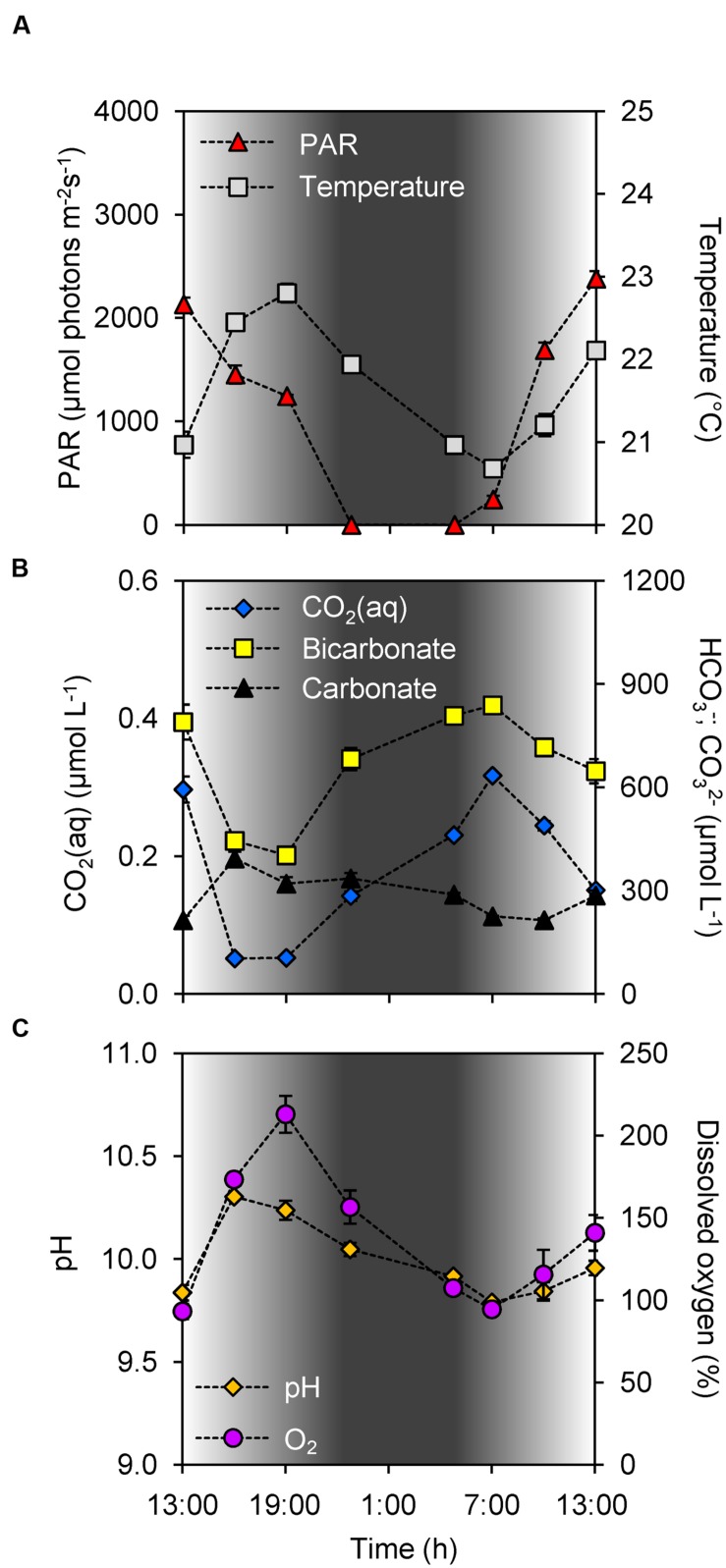
**Diel changes in environmental conditions. (A)** Photosynthetically active radiation (PAR) and water temperature. **(B)** Dissolved CO_2_ (CO_2_(aq)), bicarbonate and carbonate concentrations. **(C)** pH and dissolved oxygen. Shading indicates the light intensity measured at the water surface, with complete darkness between 22:30 and 5:00. Each data point shows the mean ± SD of three independent measurements, for photosynthetically active radiation (PAR) at the water surface and for the other environmental parameters at 0.2 m depth.

During the study period it was sunny and dry at daytime, and the photosynthetically active radiation (PAR) increased above 2,000 μmol photons m^-2^ s^-1^ in the early afternoon (**Figure 2A**). Sunset was at 21:51, the night was dry with clear skies, with sunrise at 5:42. Water temperature was uniformly distributed over the depth of the water column, peaked at 22.9°C during early evening, and dropped to 20.6°C in the early morning of the next day (**Figure 2A**). The lake is only ~1 m deep, and depth profiles of chlorophyll fluorescence indicated that the cyanobacterial community was uniformly distributed over the water column. We did not observe formation of scum layers at the water surface.

According to Henry’s law, if the concentration of dissolved CO_2_ (CO_2_(aq)) in the lake would be in equilibrium with an atmospheric partial pressure of 390 ppm, one would expect a CO_2_(aq) concentration of ~15 μmol L^-1^. Instead, however, the CO_2_(aq) concentration was depleted to 0.05 μmol L^-1^ (which is equivalent to a partial pressure of 1 ppm) during daytime, and increased during the night to a maximum of only 0.32 μmol L^-1^ (8 ppm) at the early morning of the next day (**Figure 2B**). Hence, the lake was strongly CO_2_ undersaturated, even at night. Concomitant with the diel CO_2_(aq) fluctuations, the bicarbonate concentration showed large diel variation, from ~400 μmol L^-1^ in the early evening to ~850 μmol L^-1^ in the early morning of the next day (Pearson correlation of CO_2_(aq) vs. bicarbonate: ρ = 0.94, *n* = 8, *p* < 0.01; **Figure 2B**).

CO_2_ depletion by the cyanobacterial bloom led to an increase in pH with values up to 10.3 in the afternoon [Pearson correlation of pH vs log CO_2_(aq): ρ = -0.99, *n* = 8, *p* < 0.001; **Figures 2B,C**). The pH decreased <0.2 units over depth, indicating that this shallow lake was well mixed with nearly uniform C_i_ concentrations over the depth of the lake. CO_2_ depletion was correlated with oxygen production by the bloom (Pearson correlation of CO_2_(aq) vs. dissolved oxygen: ρ = -0.95, *n* = 8, *p* < 0.001; **Figures 2B,C**), with dissolved oxygen peaking at ~200% during the early evening. During most of the day the lake was supersaturated with oxygen, except for the early morning when it was slightly undersaturated. The sodium concentration in the lake was 12.7 ± 0.4 mmol L^-1^.

### Diel Variation in Gene Expression

**FIGURE 3:**
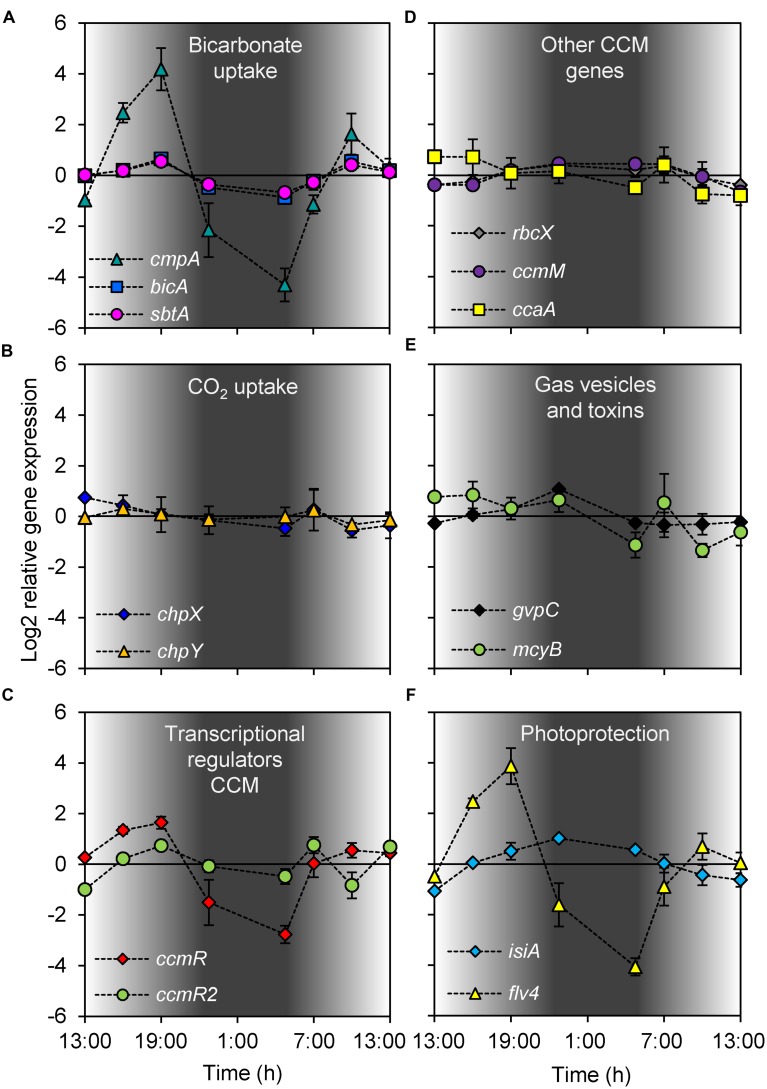
**Diel changes in gene expression. (A)** Bicarbonate uptake genes *cmpA*, *bicA*, and *sbtA*. **(B)** CO_2_ uptake genes *chpX* and *chpY*. **(C)** CCM transcriptional regulator genes *ccmR* and *ccmR2*. **(D)** RuBisCO chaperone gene *rbcX*, carboxysome gene *ccmM* and carbonic anhydrase gene *ccaA*. **(E)** Gas vesicle gene *gvpC* and microcystin gene *mcyB*. **(F)** Photoprotection genes *isiA* and *flv4*. Gene expression was quantified as log2 ratios of the expression at the given time point relative to the mean expression over the 24-h period. Shading indicates the light intensity measured at the water surface, with complete darkness between 22:30 and 5:00. Each data point shows the mean ± SD of three independent lake samples. Significant differences in gene expression between time points are reported in **Table 1**.

**FIGURE 4:**
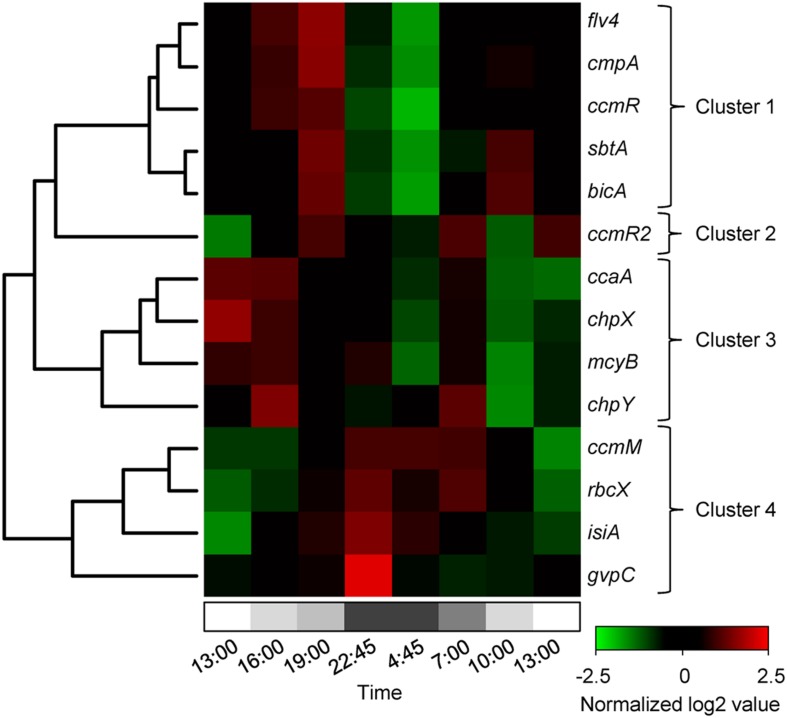
**Hierarchical clustering of the diel changes in gene expression.** The heatmap shows normalized expression data. The log2 expression values of each gene were normalized with respect to the mean and standard deviation for that gene over the 24-h period. Upregulated genes are in red and downregulated genes in green. Gray shading at the bottom indicates the light intensity at the water surface, with complete darkness between 22:30 and 5:00. Hierarchical clustering resulted in four distinct gene clusters.

Most of the investigated genes of *Microcystis* showed significant diel variation in gene expression (**Figure 3**; **Table 1**). Hierarchical cluster analysis revealed that the expression patterns of the studied genes could be grouped into four distinct clusters (**Figure 4**).

**Table 1:** Testing for significant differences in gene expression between time points.

Gene	df_1_, df_2_	*F*	*p*	Time points							
				13:00 (day 1)	16:00 (day 1)	19:00 (day 1)	22:45 (day 1)	4:45 (day 2)	7:00 (day 2)	10:00 (day 2)	13:00 (day 2)
*cmpA*	7, 16	53.808	0.000	bc	ef	f	b	a	bc	de	cd
*bicA*	7, 16	34.031	0.000	cd	de	f	ab	a	bc	ef	cde
*sbtA*	7, 16	23.886	0.000	bcd	de	e	ab	a	abc	de	cd
*chpX*	7, 16	1.620	0.200	a	a	a	a	a	a	a	a
*chpY*	7, 16	0.990	0.473	a	a	a	a	a	a	a	a
*ccmR*	7, 16	36.693	0.000	cd	de	e	b	a	c	cde	cd
*ccmR2*	7, 16	22.693	0.000	a	bc	c	b	ab	c	a	c
*rbcX*	7, 16	7.987	0.000	a	ab	bc	c	bc	c	abc	a
*ccmM*	7, 16	7.940	0.000	ab	ab	bc	c	c	c	abc	a
*ccaA*	7, 16	4.870	0.004	b	b	ab	ab	ab	ab	a	a
*gvpC*	7, 16	10.850	0.000	a	a	a	b	a	a	a	a
*mcyB*	7, 16	7.317	0.001	c	c	bc	c	ab	c	a	abc
*flv4*	7, 16	60.401	0.000	bcd	e	e	b	a	bc	d	cd
*isiA*	7, 16	22.980	0.000	a	bc	cd	d	cd	bc	ab	ab

*This table tests differences in gene expression between time points, for the data shown in **Figure 3**. The first four columns in the table indicate the genes, the degrees of freedom (df_1_ and df_2_), the value of the *F*-statistic (*F*), and the corresponding probability (*p*) of a one-way analysis of variance. The subsequent columns indicate significant differences in gene expression between time points based on Tukey’s HSD *post hoc* test (α = 0.05). Time points that do not share the same letter are significantly different from each other.*

Expression of the genes in cluster 1 increased during daytime and decreased at night (**Figure 4**). This cluster comprised the three bicarbonate uptake genes *cmpA*, *bicA*, and *sbtA*, the transcriptional regulator gene *ccmR* and the flavodiiron protein gene *flv4*. Expression of *cmpA*, encoding for one of the subunits of the bicarbonate uptake system BCT1, showed the largest diel variation in this cluster. Its maximum expression during daytime was almost 360-fold higher (log2 difference of 8.5) than its minimum expression at night (**Figure 3A**). Expression of *flv4* and *ccmR*, which is most likely a transcriptional regulator of the *cmpABCD* operon of *Microcystis* (Sandrini et al., 2015b), also showed large diel variation (**Figures 3C,F**). Expression of *bicA* and *sbtA*, encoding for the two sodium-dependent bicarbonate uptake systems, varied in tandem but at a lower amplitude than the other genes in this cluster (**Figure 3A**).

Cluster 2 consisted only of *ccmR2* (**Figure 4**), a transcriptional regulator of the *bicA*-*sbtA* operon of *Microcystis* (Sandrini et al., 2014). Similar to the genes in cluster 1, the expression of *ccmR2* increased during daytime and decreased at night, although at a lower amplitude than *ccmR* (**Figure 3C**). Its expression pattern differed from the genes in cluster 1 by a dip during the morning of the 2nd day.

Cluster 3 comprised the genes *chpX* and *chpY* (encoding the hydration subunits of the low-affinity and high-affinity CO_2_ uptake system, respectively), *ccaA* (encoding the carboxysomal carbonic anhydrase) and *mcyB* (encoding a microcystin synthetase; **Figure 4**). The cluster analysis suggests that these genes displayed a higher expression during the first daytime than during the second daytime period. However, diel variation of these genes was only minor and in most cases not significant, except for *mcyB* which decreased at night and increased briefly but significantly at first daylight of the 2nd day (**Figure 3**; **Table 1**).

Expression of the genes in cluster 4 was lowest during daytime and highest at night (**Figures 3** and **4**). This cluster comprised the CCM genes *rbcX* located in the RuBisCO operon and *ccmM* encoding for a carboxysomal shell protein, the gas vesicle protein gene *gvpC* and the gene *isiA* encoding for the iron-starvation induced protein.

### Comparison of Gene Expression Patterns with Environmental Conditions

**FIGURE 5:**
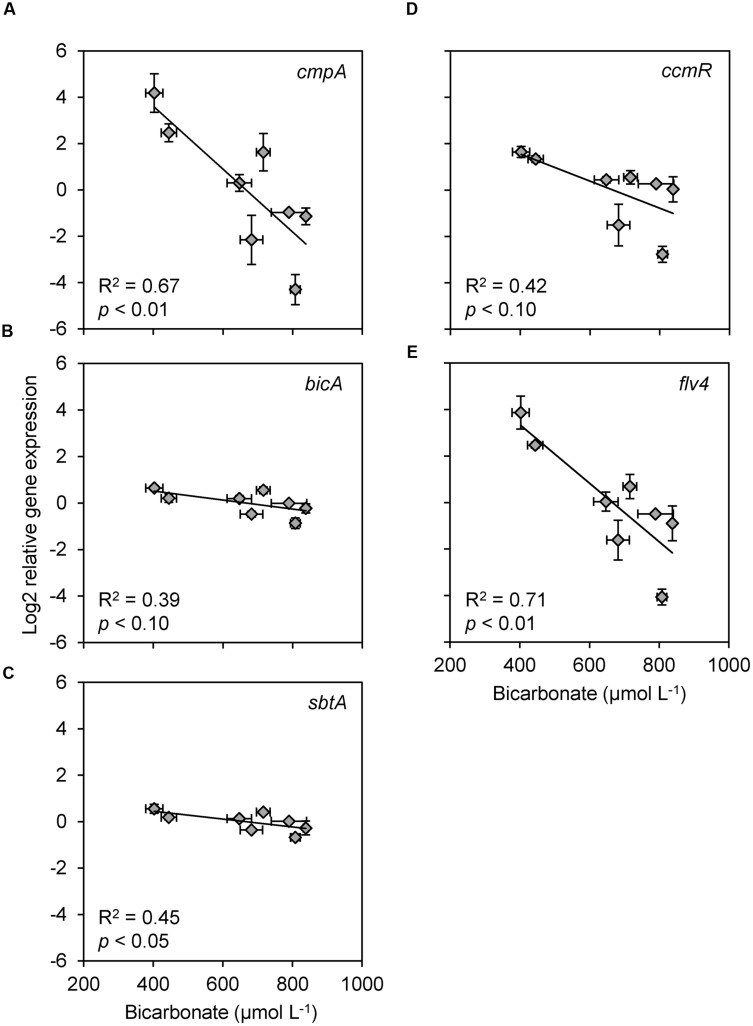
**Scatter plots of relative gene expression versus bicarbonate concentration, for all genes that showed a significant or marginally significant relationship with bicarbonate. (A–C)** Bicarbonate uptake genes **(A)** *cmpA*, **(B)** *bicA*, and **(C)** *sbtA*. **(D)** CCM transcriptional regulator gene *ccmR*. **(E)** Photoprotection gene *flv4*. Each data point shows the mean ± SD of three independent measurements. The trend lines are based on linear regression (*n* = 8).

Several of the gene expression patterns were associated with diel changes in environmental conditions (**Table 2**). Correlations with the bicarbonate concentration and incident light intensity showed the most consistent response across the gene clusters. The expression of all five genes in cluster 1 showed a significant (*p* < 0.05) or marginally significant (*p* < 0.10) negative correlation with the bicarbonate concentration, whereas none of the other gene expression patterns were correlated with bicarbonate (**Figure 5**; **Table 2**). Of these five gene expression patterns, *cmpA* and *flv4* were strongly correlated with the bicarbonate concentration, whereas *bicA*, *sbtA*, and *ccmR* showed a weaker correlation.

**Table 2:** Pearson correlation coefficients between gene expression patterns and environmental variables (*n* = 8).

Gene	CO_2_(aq)	Bicarbonate^§^	pH	Temperature	Light^§^	Dissolved oxygen
**Cluster 1:**						
*cmpA*	ns	-0.82**	ns	0.69*	ns	0.68*
*bicA*	ns	-0.62^#^	ns	ns	0.69*	ns
*sbtA*	ns	-0.67*	ns	ns	0.70*	ns
*ccmR*	ns	-0.65^#^	ns	ns	0.67*	ns
*flv4*	-0.62^#^	-0.84**	ns	0.72*	ns	0.70*
**Cluster 2:**						
*ccmR2*	ns	ns	ns	ns	ns	ns
**Cluster 3:**						
*chpX*	ns	ns	ns	ns	ns	ns
*chpY*	ns	ns	ns	ns	ns	ns
*ccaA*	ns	ns	ns	ns	ns	ns
*mcyB*	ns	ns	ns	ns	ns	ns
**Cluster 4:**						
*rbcX*	ns	ns	ns	ns	-0.92**	ns
*ccmM*	ns	ns	ns	ns	-0.94**	ns
*isiA*	ns	ns	ns	ns	-0.83**	ns
*gvpC*	ns	ns	ns	ns	ns	ns

^#^*p* < 0.10 (two-tailed), *p < 0.05 (two-tailed), **p < 0.01 (two-tailed); ns, not significant (*p* > 0.10). ^§^ Scatterplots of gene expression vs. bicarbonate concentration and incident light intensity are shown in **Figures 5** and **6**.

**FIGURE 6:**
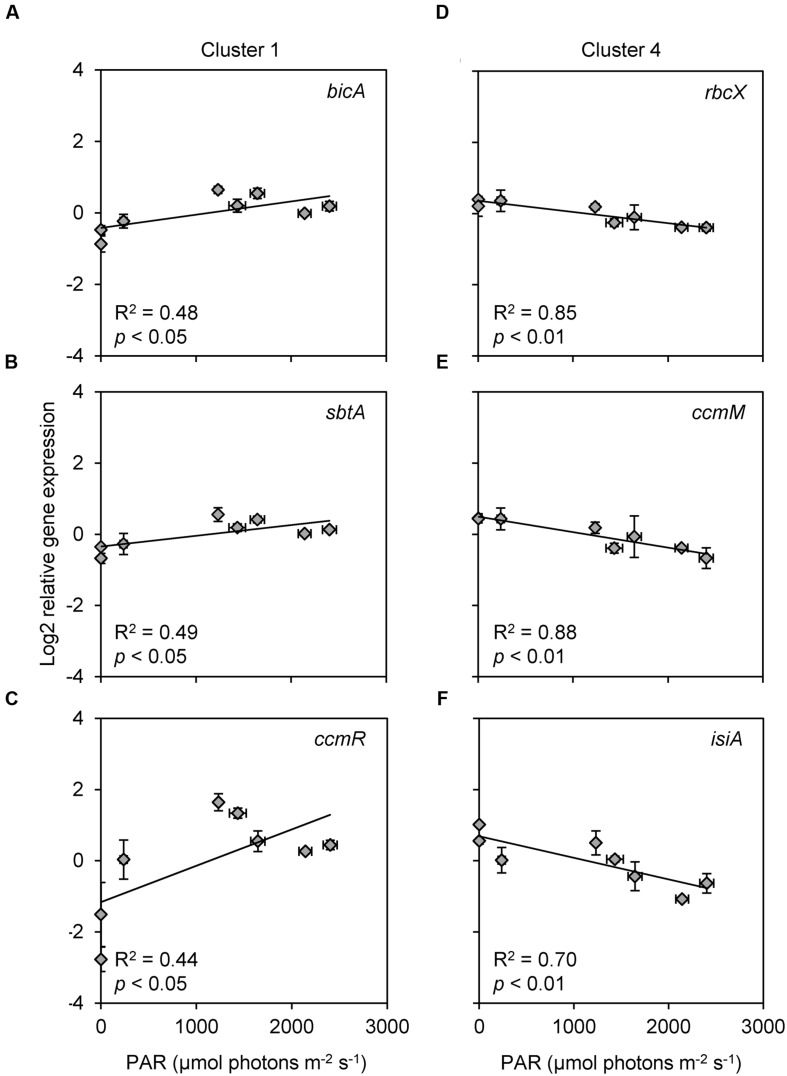
**Scatter plots of relative gene expression versus light intensity, for all genes that showed a significant or marginally significant relationship with light. (A,B)** Bicarbonate uptake genes **(A)** *bicA* and **(B)** *sbtA*. **(C)** CCM transcriptional regulator gene *ccmR*. **(D)** RuBisCO chaperone gene *rbcX*. **(E)** Carboxysomal gene *ccmM*. **(F)** Photoprotection gene *isiA*. Light intensity is indicated as PAR at the water surface. Each data point shows the mean ± SD of three independent measurements. The trend lines are based on linear regression (*n* = 8).

Expression of *bicA*, *sbtA*, and *ccmR* (cluster 1) showed a weak but significant positive correlation with the incident light intensity, whereas expression of *rbcX*, *ccmM*, and *isiA* (cluster 4) showed a significant negative correlation with the incident light intensity (**Figure 6**; **Table 2**). Furthermore, the expression of several genes (*cmpA*, *bicA*, *ccmR*, *ccmR2*, *mcyB*, and *flv4*) increased significantly from 4:45 to 7:00 AM, i.e., at first daylight (**Figure 3**; **Table 1**) before the C_i_ concentration was depleted by the bloom (**Figure 2B**).

## Discussion

### Inorganic Carbon Dynamics

Most lakes worldwide are supersaturated with CO_2_ in the absence of phytoplankton blooms, because of, e.g., mineralization of organic carbon produced in the lake and received from the surrounding watershed (Cole et al., 1994; Sobek et al., 2005; Lazzarino et al., 2009). For instance, in a comparative study across almost 5,000 lakes, Sobek et al. (2005) found a mean pCO_2_ of 1,287 ppm [equivalent to a CO_2_(aq) concentration of ~50 μmol L^-1^ according to Henry’s law]. However, as our lake study illustrates, dense phytoplankton blooms in eutrophic lakes can deplete the CO_2_(aq) concentration and increase pH, and bring about substantial diel variation in the concentrations of dissolved inorganic carbon (DIC) and oxygen. During daytime, the CO_2_(aq) concentration was reduced to only 0.05 μmol L^-1^ (1 ppm) and pH increased above 10. At night, the CO_2_(aq) concentration increased, but the lake remained severely undersaturated. Similar observations have been made in other lakes with dense phytoplankton blooms. The photosynthetic activity of dense blooms can go up to 12.5–50 μmol C L^-1^ h^-1^ (Hein, 1997), which is sufficient to deplete the CO_2_(aq) concentration in lakes within a few hours (Talling, 1976; Maberly, 1996). Balmer and Downing (2011) reported several eutrophic lakes in which the CO_2_(aq) concentration was drawn down to less than 0.1 μmol L^-1^ during daytime, comparable to the low concentrations measured in our study. One limitation of our study is that we investigated only a single 24-h period, but other studies monitored the CO_2_ dynamics in lakes over longer time spans. Maberly (1996) monitored the inorganic carbon concentrations of Lake Esthwaite Water (UK) for an entire year, and found large diel fluctuations in the CO_2_(aq) concentration and pH (sometimes up to 1.8 units) throughout the summer. During dense phytoplankton blooms, CO_2_(aq) concentrations in Lake Esthwaite remained undersaturated with respect to the atmospheric CO_2_ level for several consecutive weeks. Verspagen et al. (2014) monitored Lake Volkerak (The Netherlands) on a biweekly basis for two consecutive years, during winter, the lake was supersaturated with CO_2_. In summer and early fall, however, Lake Volkerak was covered by dense blooms of the cyanobacterium *Microcystis*, and the lake became undersaturated with CO_2_ for several months.

Depletion of the CO_2_(aq) concentration by dense phytoplankton blooms creates a pCO_2_ gradient across the air-water interface, which implies that these lakes act as a sink for atmospheric CO_2_ (Balmer and Downing, 2011). More specifically, the CO_2_ influx (g_CO2_) from the atmosphere into a lake can be estimated from the difference between the expected concentration of CO_2_(aq) in equilibrium with the atmosphere (according to Henry’s law) and the measured concentration of CO_2_(aq) (Siegenthaler and Sarmiento, 1993; Cole et al., 2010):

(1) $${gco}_{2}=\;v\left(K_{H}{pCO}_{2}-{CO}_{2}\left(aq\right)\right)$$

where *v* is the gas transfer velocity across the air-water interface, *K*_H_ is the solubility constant of CO_2_ gas in water (*K*_H_ = 0.0375 mol L^-1^ atm^-1^ at 21.5°C; Weiss, 1974), and pCO_2_ is the partial pressure of CO_2_ in the atmosphere. Assuming a typical gas transfer velocity of *v* = 0.02 m h^-1^ (Crusius and Wanninkhof, 2003; Cole et al., 2010), an atmosphere with a pCO_2_ of 390 ppm and an average CO_2_(aq) of 0.2 μmol L^-1^ (**Figure 2B**), the daily CO_2_ influx into Lake Kennemermeer would amount to ~7 mmol m^-2^ d^-1^. This is a substantial CO_2_ influx, which will fuel further development of the cyanobacterial bloom. Moreover, the above calculation indicates that a doubling of the atmospheric CO_2_ concentration to 780 ppm will cause a near-doubling of the CO_2_ influx into the lake to ~14 mmol m^-2^ d^-1^, if we assume that dense cyanobacterial blooms still deplete the dissolved CO_2_ concentration. These results are in line with model predictions that rising atmospheric CO_2_ concentrations will stimulate cyanobacterial blooms in eutrophic lakes (Schippers et al., 2004; Verspagen et al., 2014; Visser et al., 2016).

Owing to the high pH induced by the cyanobacterial bloom, bicarbonate and carbonate were the most abundant inorganic carbon species in Lake Kennemermeer, whereas the CO_2_(aq) concentration was more than three orders of magnitude lower. The amplitude of the daily variation in CO_2_(aq) concentration was less than 0.3 μmol L^-1^, whereas the bicarbonate concentration varied more than 400 μmol L^-1^ during the 24-h period. We note that *Microcystis* often forms colonies. Colony morphology may affect gas transfer inside the colony, and cells deep within the colony may experience other CO_2_ and bicarbonate concentrations than cells at the outer edge of the colony. Nevertheless, given the low CO_2_(aq) concentration in the ambient lake water, it seems most likely that colonial cyanobacteria in these dense blooms will be strongly dependent on bicarbonate uptake to cover the high C demands of their photosynthetic activity, and may adapt to the large diel fluctuations in bicarbonate availability by adjusting the production of their different bicarbonate uptake systems.

### Co-occurrence of C_i_ Uptake Genotypes

The phytoplankton bloom in Lake Kennemermeer consisted of a mixture of cyanobacterial species, including Pseudanabaenaceae, *A. hungarica*, small Chroococcales and *Microcystis* spp. For most of these species, genome sequences are not available, and we could not develop suitable primers to target their CCM genes. Therefore, we restricted our analysis to *Microcystis*, for which extensive sequence information is available (Kaneko et al., 2007; Frangeul et al., 2008; Humbert et al., 2013; Sandrini et al., 2014). For the interpretation of the results, however, it is important to realize that *Microcystis* does not grow in isolation but competes for C_i_ and other potentially limiting resources with other species in the lake. Changes in the relative abundances of C_i_ uptake genotypes and CCM gene expression of *Microcystis* are related to C_i_ drawdown and CO_2_ production by the entire bloom community.

Earlier we reported that *Microcystis* isolates from Lake Pehlitzsee (Germany) and Lake Volkerak (The Netherlands) contained both *sbtA* and *bicA* + *sbtA* strains, suggesting that these two C_i_ uptake genotypes may coexist (Sandrini et al., 2014). Our current results show that each of the three C_i_ uptake genotypes of *Microcystis* were present in Lake Kennemermeer with relative abundances of ≥15%, demonstrating that all three genotypes can co-occur in the same lake.

In a companion study, we monitored the population dynamics of the C_i_ uptake genotypes of *Microcystis* in Lake Kennemermeer from June to October 2013 (Sandrini, 2016). The results showed that *bicA* + *sbtA* strains dominated during the dense summer bloom, whereas *sbtA* strains and *bicA* strains were present at lower abundances, as reported here. However, *bicA* + *sbtA* strains were replaced by *bicA* strains when C_i_ concentrations increased during the demise of the cyanobacterial bloom in autumn. The trade-off between affinity and flux rate of the bicarbonate uptake systems provides a parsimonious explanation for these results. The SbtA enzyme has a high affinity for bicarbonate but low flux rate, whereas the BicA enzyme has a low affinity but high flux rate (Price, 2011). The dominance of *bicA* + *sbtA* strains, containing both bicarbonate uptake systems, indicates that strains that can cope with strong diel fluctuations of the bicarbonate concentration may have a selective advantage during the summer bloom. Indeed, laboratory experiments showed that *bicA* + *sbtA* strains can grow well across a wide range of C_i_ concentrations (Sandrini et al., 2014). The subsequent replacement of *bicA* + *sbtA* strains by *bicA* strains indicates that the high-affinity uptake system SbtA becomes superfluous when the C_i_ availability increases in autumn, providing a selective advantage for strains with only the high-flux uptake system BicA at high C_i_ levels.

### Expression of C_i_ Uptake Genes

In contrast to most laboratory studies, our gene expression data reflect the average gene expression over the entire *Microcystis* population rather than the expression of a single strain. Nevertheless, our lake study shows distinct diel patterns of gene expression. In cyanobacteria, the expression of many genes is under the control of the circadian clock genes *kaiA*, *kaiB*, and *kaiC* (Ishiura et al., 1998; Iwasaki et al., 2002), and it is difficult to disentangle the role of environmental variation versus an internal circadian clock based on field observations alone. However, *kai*-mutants of the cyanobacterium *Synechococcus* PCC 7002 have shown that several CCM genes are *kai*-independent cycling genes (Ito et al., 2009). Therefore, it is plausible that the CCM genes of *Microcystis* are *kai*-independent as well. Our field data show that diel changes in the expression of the C_i_ uptake genes were associated with diel variation in C_i_ and/or light availability. This is in agreement with many laboratory experiments, which have demonstrated that the expression of cyanobacterial CCM genes can be strongly affected by changes in C_i_ and light availability (Hihara et al., 2001; McGinn et al., 2003; Woodger et al., 2003; Wang et al., 2004; Schwarz et al., 2011; Burnap et al., 2013; Sandrini et al., 2015a). We will therefore focus our interpretation of the expression data on diel variation in these environmental factors.

The expression of *cmpA*, encoding for a subunit of the high-affinity bicarbonate transporter BCT1, was negatively correlated with the bicarbonate concentration in the lake (**Figure 5A**). This pattern is in agreement with laboratory studies of *Microcystis* and other cyanobacteria, where *cmpA* expression was also strongly down-regulated at elevated C_i_ conditions (Sandrini et al., 2015a,b), and up-regulated under C_i_-limiting conditions (Woodger et al., 2003; Wang et al., 2004; Schwarz et al., 2011). Bicarbonate concentrations in the lake decreased at daytime and increased at night, which likely caused the high *cmpA* expression in the late afternoon and a low *cmpA* expression at the end of the night. The large amplitude of the diel changes in *cmpA* expression suggests that the BCT1 enzyme is largely degraded at night and resynthesized each day. Expression of *cmpA* was not significantly correlated with light availability. However, bicarbonate uptake by BCT1 is ATP-dependent and ATP synthesis is driven by the light reactions of photosynthesis, which may explain the increase in *cmpA* expression at first daylight when the bicarbonate concentration was still high (**Figures 2B** and **3A**). This explanation is also in agreement with previous laboratory studies under C_i_-limited conditions, which showed increased *cmpA* expression at elevated light levels in several other cyanobacteria (Woodger et al., 2003; McGinn et al., 2004). Yet, ATP itself is probably not a regulator of the *cmpABCD* operon (Burnap et al., 2015). More likely, regulatory metabolites such as NADP^+^, α-ketoglutarate (α-KG), 2-phosphoglycolate (2PG) and ribulose-1,5-bisphosphate (RuBP), of which the levels can be affected by both light and C_i_ levels, control the activity of the CCM transcriptional regulators that in turn regulate the expression of the C_i_ uptake genes.

Expression of the bicarbonate uptake genes *bicA* and *sbtA* was also negatively correlated with the bicarbonate concentration (**Figures 5B,C**). In addition, their expression was positively correlated with the incident light intensity (**Figures 6A,B**). The expression of *bicA* and *sbtA* fluctuated in tandem (**Figure 3A**), likely because the *Microcystis* population was dominated by *bicA* + *sbtA* strains, in which both genes are located on the same operon (Sandrini et al., 2014). However, the diel variation in expression of *bicA* and *sbtA* had a much smaller amplitude than *cmpA*. The sodium concentration in the lake (12.7 ± 0.4 mmol L^-1^) allowed near-maximum activity of the sodium-dependent bicarbonate uptake systems BicA and SbtA, since both uptake systems have half-saturation constants of 1–2 mmol L^-1^ sodium (Price et al., 2004; Du et al., 2014). Therefore, the sodium concentration is unlikely to limit the amplitude of *bicA* and *sbtA* expression. Laboratory studies have shown that the response of *bicA* and *sbtA* expression to changes in C_i_ availability varies among *Microcystis* strains (Sandrini et al., 2015b). In some strains, *bicA* and *sbtA* expression is strongly down-regulated at elevated C_i_ conditions, whereas in other strains these genes are constitutively expressed. Hence, whether the observed low-amplitude variation in our field data reflects a generic pattern for many *Microcystis* blooms or a specific pattern for the *Microcystis* populations in our lake study remains to be investigated. Price et al. (2013) suggested that BicA and SbtA are simply inactivated by darkness and remain safeguarded for renewed activity at dawn, which may explain the low amplitude variation in gene expression, although the exact mechanisms of inactivation and re-activation remain to be revealed. Indeed, recently it was shown that SbtB is involved in the inhibition of SbtA during darkness (Du et al., 2014).

The *chpX* and *chpY* genes, encoding hydration subunits of the low-affinity and high-affinity CO_2_ uptake system, respectively, were constitutively expressed in our lake study (**Figure 3B**). Expression of these genes was not correlated with diel variation in CO_2_(aq) concentration or light intensity (**Table 2**). A possible reason could be that the diel variation in CO_2_(aq) concentrations was too low (from 0.05 to 0.32 μmol L^-1^) to affect expression of the CO_2_ uptake genes. However, both CO_2_ uptake genes were also constitutively expressed in laboratory studies with axenic *Microcystis* strains exposed to a much larger increase in CO_2_(aq) concentration (Sandrini et al., 2015a,b). In other cyanobacterial species, constitutive expression was established for the low-affinity CO_2_ uptake gene *chpX* whereas expression of the high-affinity CO_2_ uptake gene *chpY* was induced at low C_i_ levels (Woodger et al., 2003; Wang et al., 2004; Eisenhut et al., 2007; Schwarz et al., 2011). Furthermore, Jensen et al. (2011) found upregulation of both *chpY* and *chpX* during daytime for a *Synechococcus* mat. Hence, the CO_2_ uptake genes of different species respond differently to changing environmental conditions.

Expression of the CCM transcriptional regulator gene *ccmR* was negatively correlated with the bicarbonate concentration, similar to the bicarbonate uptake genes (**Figure 5D**). *Microcystis* lacks the transcriptional regulator gene *cmpR*, which regulates expression of the *cmpABCD* operon in some other cyanobacteria (Omata et al., 2001). Instead, CcmR most likely regulates expression of the *cmpABCD* operon in *Microcystis* (Sandrini et al., 2014, 2015b), which explains the similar gene expression patterns of *cmpA* and *ccmR* in our study. The *ccmR2* gene is located directly upstream of the *bicA/sbtA* operon, and appears to be involved in the transcription of this operon (Sandrini et al., 2014). This gene formed a separate cluster in our analysis (**Figure 4**), probably because of a single deviant data point at 10:00 AM. Omitting this single data point, *ccmR2* showed similar diel variation in expression as the bicarbonate uptake genes and *ccmR* (**Figures 3A,C**).

### Diel Changes in Expression of Other Genes

The flavodiiron protein gene *flv4*, which is in one operon with *flv2*, was assigned to the same cluster as the bicarbonate uptake genes and the regulator gene *ccmR* (**Figure 4**). Flavodiiron proteins are stress proteins known to be involved in the acclimation to low C_i_ conditions (Zhang et al., 2009; Allahverdiyeva et al., 2011; Bersanini et al., 2014; Sandrini et al., 2015a), and can also be up-regulated by high light (Zhang et al., 2009). Similarly, in our study *flv4* expression was increased at first daylight and reached high levels when C_i_ was depleted in the afternoon (**Figure 3F**). In contrast, Straub et al. (2011) found no significant diel variation in the expression of *flv2* and *flv4* in laboratory experiments with the axenic *Microcystis* strain PCC 7806, probably because their experiments were sparged with 1% CO_2_ and hence C_i_ concentrations were not limiting in their study. Both genes, *flv2* and *flv4*, were shown to be involved in photoprotection of the PSII complex (Zhang et al., 2009). Hence, our study indicates that Flv2/4 proteins are highly active in daytime photoprotection of cyanobacterial blooms.

Interestingly, the iron-stress chlorophyll-binding protein IsiA is also involved in photoprotection (Havaux et al., 2005), but the expression pattern of *isiA* was very different from *flv4* (**Figure 3F**). Expression of *isiA* was high at night but low during daytime (**Figure 3F**). In addition to its photoprotective function, IsiA is also involved in the formation of PSI antennae enhancing the light harvesting ability and in the storage of free chlorophyll molecules (Yeremenko et al., 2004; Havaux et al., 2005). Possibly, the turnover of photosystems and presence of free chlorophylls at night might stimulate *isiA* expression for temporary chlorophyll storage.

Expression of the *rbcX*, *ccmM*, *ccaA*, *gvpC*, and *mcyB* genes showed only minor diel variation. The RuBisCO gene *rbcX* and carboxysomal gene *ccmM* were negatively correlated with light intensity (**Figures 6D,E**), which could indicate that cells slightly increase the RuBisCO and carboxysome numbers at night to prepare for cell division, although it was previously shown that *Microcystis* cell division occurs both during daytime and at night (Yamamoto and Tsukada, 2009). Alternatively, the negative correlation might reflect an afternoon photosynthesis dip due to photoinhibition (Ibelings and Maberly, 1998; Wu et al., 2011) when the incident light intensity exceeded 1,500 μmol photons m^-2^ s^-1^ (**Figure 2A**). Expression of the other three genes (*ccaA*, *gvpC*, and *mcyB*) did not reveal a significant correlation with any of the environmental variables investigated (**Table 2**).

## Conclusion

Cyanobacteria are often assumed to be favored at low C_i_ and high pH conditions, because of the presence of an effective CCM (e.g., Shapiro, 1997). However, recent laboratory studies revealed considerable genetic and phenotypic diversity in the CCM of the ubiquitous harmful cyanobacterium *Microcystis* (Sandrini et al., 2014, 2015b). Some strains perform well at low C_i_ levels, whereas other strains are much better competitors under high C_i_ conditions, suggesting that it might be difficult to foretell how natural mixtures of different *Microcystis* strains will respond to changes in C_i_ availability. Yet, our lake study showed consistent patterns in gene expression. The bicarbonate concentration in the lake showed large diel fluctuations, which may explain the predominance of *bicA* + *sbtA* strains and the concurrent diel fluctuations in expression of the bicarbonate uptake genes and their regulator genes. Hence, we conclude that the genetic and phenotypic versatility of the CCM of *Microcystis* enables a remarkably flexible but coherent response to the large diel fluctuations in C_i_ conditions often encountered in dense blooms.

## Materials and Methods

### Study Area and Sampling

Lake Kennemermeer (52°27′18.5′′N, 4°33′48.6′′E) is located north-west of Amsterdam, The Netherlands, near the North Sea coast (**Figure 1**). The man-made lake has been used as bathing water, but nowadays it is no longer in use as recreational lake because of yearly recurrent problems with harmful cyanobacterial blooms. The lake has a maximum depth of ~1 m, and surface area of ~0.1 km^2^. The shallow lake is well mixed by wind throughout the year.

During a dense summer bloom in July 2013, we monitored diel variation in gene expression and environmental conditions. The blooming area spanned the entire lake and the phytoplankton biomass peaked two times that year, from end of June till the 3rd week of July and in the 1st week of September. A small boat was used to sample the north side of the lake (**Figure 1**) at 13:00, 16:00, 19:00, and 22:45 of July 17, and at 4:45, 7:00, 10:00, and 13:00 of July 18. At each time point, the incident light intensity PAR just above the water surface, and vertical profiles of light intensity, water temperature, pH, dissolved oxygen and chlorophyll fluorescence were measured in triplicate at depth intervals of 0.1 m using a Hydrolab Surveyor with a Datasonde 4a (OTT Hydromet, Loveland, CO, USA). Furthermore, three independent water samples of 5 L each were collected at 0.2 m depth using plastic 10 L tanks. The samples were processed immediately for further analyses.

### Cell Counts

Lugol’s iodine was added to 40 mL aliquots of fresh lake samples (1:100 v/v of a 5% solution) to preserve phytoplankton cells for microscopy. The samples were stored at 4°C until identification. Phytoplankton was counted according to the Utermöhl-method adjusted to the European standard protocol NEN-EN 15204 using a Lyca DM IRB inverted light microscope (Lyca Microsystems BV, Rijswijk, The Netherlands). Phytoplankton was identified to the genus level, and if possible to the species level. Biovolume was estimated from cellular dimensions and geometry (Hillebrand et al., 1999). Individual *Microcystis* cells were counted after disintegrating the colonies with KOH (Kardinaal et al., 2007).

### Dissolved Inorganic Carbon and Sodium

For analysis of the DIC and sodium concentration, lake samples were filtered on site using 1.2 μm pore size 47 mm GF/C filters (Whatman GmbH, Dassel, Germany) followed by 0.45 μm pore size 47 mm polyethersulfone membrane filters (Sartorius AG, Goettingen, Germany). The filtrate was transferred to sterile plastic urine analysis tubes (VF-109SURI; Terumo Europe N.V., Leuven, Belgium) which were filled completely (with an inserted needle to release all air), and stored at 4°C until further analysis. A TOC-V_CPH_ TOC analyzer (Shimadzu, Kyoto, Japan) was used to determine the DIC concentration, with 3–5 technical replicates per sample. Concentrations of dissolved CO_2_ (CO_2_(aq)), bicarbonate and carbonate were calculated from DIC and the pH and temperature of the lake (Stumm and Morgan, 1996). Sodium ion concentrations were measured using an Optima 8000 ICP-OES Spectrometer (Perkin Elmer, Waltham, MA, USA).

### RNA Extraction

For RNA extraction, lake samples were filtered on-site with large 90 mm GF/C filters (Whatman GmbH, Dassel, Germany; 1.2 μm pore size) to concentrate phytoplankton biomass. Next, the 90 mm filters were dissected into several pieces using sterile surgical blades, which were transferred to 2 mL tubes that were filled with 1 mL TRIzol (Thermo Fisher Scientific, Waltham, MA, USA). The tubes were shaken vigorously, transported to the lab in a CX 100 Dry Shipper (Taylor Wharton, Theodore, AL, USA), and stored at -80°C until further analysis. RNA was extracted using 0.5 mm bashing beads (Zymo Research, Orange, CA, USA) to facilitate cell disruption, and purified according to Sandrini et al. (2015a) with the Direct-Zol^TM^ RNA MiniPrep kit (Zymo Research, Orange, CA, USA) including in-column DNase I digestion. RNA concentrations were quantified using a Nanodrop 1000 spectrophotometer (Thermo Fisher Scientific, Waltham, MA, USA). All RNA samples had *A*_260_/*A*_280_ and *A*_260_/*A*_230_ values above 1.8.

### Primer Development

Primers were developed to study expression of the *Microcystis* genes *bicA*, *sbtA*, *cmpA*, *chpX*, *chpY*, *ccmR*, *ccmR2*, *rbcX*, *ccmM*, *ccaA*, *mcyB*, *gvpC*, *isiA*, and *flv4*, using 16S rRNA as ‘reference gene.’ The complete list of primers is shown in Supplementary Table S1. The primers were designed to match the sequences of *Microcystis* strains NIES-843, PCC 7005, PCC 7941, PCC 7806, PCC 9432, PCC 9443, PCC 9701, PCC 9717, PCC 9806, PCC 9807, PCC 9808, PCC 9809, and T1-4. The *bicA*, *sbtA* and *ccmR2* primers were also based on sequences of strains CCAP 1450/10, CCAP 1450/11, HUB 5-2-4, HUB 5-3, NICA-CYA 140, V145, and V163 (Sandrini et al., 2014). The primer design made use of IDT SciTools (Owczarzy et al., 2008) to develop primers with a similar melting temperature and GC content, such that all primers could be used under the same PCR conditions. We also used IDT SciTools to avoid undesirable secondary structures such as hairpins, self-dimers, or heterodimers. Furthermore, we performed primer-BLAST searches to assess the specificity of the primers (Ye et al., 2012). In this way, we ensured that the primers matched the gene sequences of *Microcystis*, but always included several mismatches with gene sequences of other cyanobacteria. The primers were tested on gDNA (obtained as described below) of 11 different *Microcystis* strains to ensure one target PCR product was formed. These PCR reactions were done with the GoTaq^®^Hot Start Polymerase kit (Promega Corporation, Madison, WI, USA) according to the supplier’s instructions. After an initial denaturation of 2 min at 95°C, 35 cycles were used that consisted of a denaturation step at 95°C for 45 s, an annealing temperature step at 60°C for 30 s and an extension step at 72°C for 3 min. Subsequently, a final extension step at 72°C was used for 5 min. The reactions contained 0.3 μmol L^-1^ primers and 10 ng gDNA in a total reaction volume of 25 μL. Other reaction components were added as instructed by the supplier. Gel electrophoresis showed that only the targeted gDNA sequences were amplified (no by-products were detected). As a negative control, the primers were tested on gDNA of *Anabaena circinalis* CCAP 1403/18, *Aphanizomenon flos-aqua* CCAP 1401/7, and *Planktothrix agardhii* CCAP 1460/1, which did not result in PCR amplification.

### RT-qPCR Gene Expression Analysis

To quantify gene expression, cDNA was synthesized by reverse transcription of the RNA samples with Superscript III (Thermo Fisher Scientific, Waltham, MA, USA) according to Sandrini et al. (2015a). Subsequently, the qPCR Maxima^®^SYBR Green Master Mix (2x; Thermo Fisher Scientific, Waltham, MA, USA) was applied on the cDNA samples according to Sandrini et al. (2015a), in an ABI 7500 Real-Time PCR system (Applied Biosystems, Foster City, CA, USA). The two-step cycling protocol was used, with a denaturation temperature of 95°C (15 s) and a combined annealing/extension temperature of 60°C (60 s) during 40 cycles. The reactions contained 0.3 μmol L^-1^ primers and 1 μL of 10 times diluted cDNA from the RT reaction in a total reaction volume of 25 μL. Other reaction components were added as instructed by the supplier. ROX solution was used to correct for any well-to-well variation. Melting curve analysis was performed on all measured samples to rule out non-specific PCR amplification. The melting curves confirmed that only one PCR product was amplified in each run.

Amplification efficiencies of individual runs (*E*) were calculated with LinRegPCR (version 2012.3; Ramakers et al., 2003; Ruijter et al., 2009) and were between 1.8 and 2.0 (Supplementary Table S1). Application of the primer sets to gDNA from six different *Microcystis* strains showed that the primer sets amplify the target genes from the different genotypes with similar efficiencies (see Supplementary Table S1 in Sandrini et al., 2015b). Time point 0 (13:00 of the 1st day) was used as ‘reference sample,’ and 16S rRNA was used as ‘reference gene.’ Each RT-qPCR plate contained a reference sample and samples with primers targeting 16S rRNA to correct for plate effects (differences in absolute fluorescence signal between individual plates). The data were baseline corrected using LinRegPCR, and the same software was used to calculate quantification cycle (*C*q) values. LinRegPCR did not detect samples without amplification, without a plateau, with a baseline error or noise error, or with deviating amplification efficiencies. Negative control samples did not show significant amplification. Gene expression was quantified as the log2 ratio of the expression at a given time point relative to the mean expression over the 24-h period using the comparative *C*_T_ method (Livak and Schmittgen, 2001). To determine if gene expression varied significantly between time points, one-way analysis of variance (ANOVA) was used with *post hoc* comparison of the means based on Tukey’s HSD test (α = 0.05) using SPSS version 20.0.

Hierarchical clustering was applied to compare the expression patterns of the studied genes. For each gene, time series of the expression values were normalized by the transformation (*x* - μ)/σ, where *x* is the original data point, μ is the mean of the time series, and σ is its standard deviation. Thus, all genes obtained normalized expression patterns with mean 0 and standard deviation 1. Hierarchical clustering and heatmap representation of the normalized gene expression data was done using the hclust and heatmap.2 functions of the gplots package in R version 3.0.2. We used the complete linkage clustering method for hierarchical clustering (Everitt et al., 2011).

### Quantification of *Microcystis* Genotypes

To quantify the relative abundances of different *Microcystis* genotypes, we applied qPCR on purified gDNA (Supplementary Table S1). Lake samples were filtered on-site over 1.2 μm pore size 25 mm GF/C filters (Whatman GmbH, Dassel, Germany), and loaded filters were stored at -20°C. Subsequently, gDNA was extracted using the ZR Fungal/Bacterial DNA MiniPrep^TM^ kit (Zymo Research, Orange, CA, USA) and further purified using the DNA Clean and Concentrator^TM^-25 kit (Zymo Research) according to the supplier’s instructions. The gDNA samples were analyzed using a Nanodrop 1000 spectrophotometer (Thermo Fisher Scientific, Waltham, MA, USA), which resulted in *A*_260_/*A*_280_ values above 1.8 for all samples.

The Maxima^®^SYBR Green Master Mix (2x) kit (Thermo Fisher Scientific, Waltham, MA, USA) was applied to the purified gDNA according to the supplier’s instructions in an ABI 7500 Real-Time PCR system (Applied Biosystems, Foster City, CA, USA). The two-step cycling protocol was used, and the reaction settings and melting curve analysis were the same as in the RT-qPCR section (see above). The reactions contained 0.3 μmol L^-1^ primers and 10 ng gDNA from lake samples in a total reaction volume of 25 μL. For data analysis, the LinRegPCR software tool (version 2012.3; Ramakers et al., 2003; Ruijter et al., 2009) and the comparative *C*_T_ method (Livak and Schmittgen, 2001) were used.

To determine the relative abundances of the different C_i_ uptake genotypes, we used the *bicA* gene (primers bicA-F1 and bicA-R1; Supplementary Table S1) and the *sbtA* gene (primers sbtA-F1 and sbtA-R1; Supplementary Table S1) as ‘target genes.’ The *bicA* + *sbtA* gene of the *bicA* + *sbtA* strains (primers bicA-F2 and sbtA-R2; Supplementary Table S1) served as ‘reference gene.’ Purified gDNA of the axenic laboratory strains PCC 7005 and PCC 7941 (both *bicA* + *sbtA* strains; Sandrini et al., 2014) served as ‘reference samples,’ to calculate the relative ratios of (1) the *bicA* gene versus the *bicA* + *sbtA* gene, and (2) the *sbtA* gene versus the *bicA* + *sbtA* gene. We note that the *bicA* gene is present in both *bicA* strains and *bicA* + *sbtA* strains, and similarly the *sbtA* gene is present in both *sbtA* strains and *bicA* + *sbtA* strains. Hence, the relative abundances of the different C_i_ uptake genotypes can be calculated from the above two ratios based on the assumption that the sum of the *bicA* strains, *sbtA* strains and *bicA* + *sbtA* strains equals 100%.

To determine the relative abundance of potentially toxic genotypes, we used *mcyB* (primers mcyB-F and mcyB-R; Supplementary Table S1) as ‘target gene’ and the RuBisCO chaperone gene *rbcX* (primers rbcX-F and rbcX-R) present in all *Microcystis* strains as ‘reference gene.’ Purified gDNA of the axenic toxic strains PCC 7806 and PCC 7941 was used as ‘reference samples.’

## Author Contributions

GS, HM, and JH designed the study. GS, RT, and HM performed the fieldwork, assisted by JMS and SB. GS analyzed most field samples. GS, RT, HM, and JH analyzed the data. GS, HM, and JH wrote the manuscript, and all authors commented on the final version.

## Conflict of Interest Statement

The authors declare that the research was conducted in the absence of any commercial or financial relationships that could be construed as a potential conflict of interest.
