# Supplementary material for: Diel Variation in Gene Expression of the CO2-Concentrating Mechanism during a Harmful Cyanobacterial Bloom
Source: Front Microbiol. 2016 Apr 22;7:551. doi: 10.3389/fmicb.2016.00551 (PMC4840274; doi:10.3389/fmicb.2016.00551)
Supplement: Supplementary file 1 [file Table_1.DOCX]

Supplementary Material

Diel variation in gene expression of the CO_2_-concentrating mechanism during a harmful cyanobacterial bloom

Giovanni Sandrini, Robert P. Tann, J. Merijn Schuurmans, Sebastiaan A. M. van Beusekom, Hans C. P. Matthijs, Jef Huisman*

**Supplementary Table 1. Overview of *Microcystis* primer pairs used for qPCR analysis.**

| **Primer name** | **Sequence 5’🡪 3’ (length)** | **Target gene(s)** | **Function of complete protein/complex** | **Locus tag** | **Accession no. (Genbank)** | **Expected**  **product size (bp)** | **Amplification efficiency (*E*)** | **RT-qPCR on**  **cDNA (1)**  **or**  **qPCR on gDNA (2)** | **Reference** |
| --- | --- | --- | --- | --- | --- | --- | --- | --- | --- |
| 16S-F | GTCGAACGGGAATCTTCGGAT (21) | *16S rRNA* | Used as reference gene | IPF_5548 | AM778951.1 | 157 | 1.88±0.03 | 1 | Sandrini *et al.*, 2015a |
| 16S-R | GCTAATCAGACGCAAGCTCTTC (22) |  |  |  |  |  |  | 1 | Sandrini *et al.*, 2015a |
| cmpA-F | GTTAAACACCCAGGGTAACGGA (22) | *cmpA* | High-affinity ATP-dependent bicarbonate uptake system | IPF_2181 | AM778958.1 | 180 | 1.87±0.01 | 1 | Sandrini *et al.*, 2015a |
| cmpA-R | GCTAACCAGTAACGAATCCAGAAGT (25) |  |  |  |  |  |  | 1 | Sandrini *et al.*, 2015a |
| bicA-F1 | CAAGCTAACGGTCGCATCAT (20) | *bicA* | Low-affinity bicarbonate/sodium symporter | IPF_4911 | AM778949.1 | 132 | 1.89±0.01 | 1,2 | Sandrini *et al.*, 2015a |
| bicA-R1 | AGGCACATCACTCAAGTCCA (20) |  |  |  |  |  |  | 1,2 | Sandrini *et al.*, 2015a |
| sbtA-F1 | CTGGCCTTTTTGATTGGTGG (20) | *sbtA* | High-affinity bicarbonate/sodium symporter | MAE_62090 | AP009552.1 | 143 | 1.89±0.02 | 1,2 | Sandrini et al., 2015b |
| sbtA-R1 | AGGTTGGAATTGCGGATGG (19) |  |  |  |  |  |  | 1,2 | Sandrini et al., 2015b |
| bicA-F2 | TCAAGACCCATCCTCACCA (19) | *bicA* | Low-affinity bicarbonate/sodium symporter | IPF_4911 | AM778949.1 | 264 | 1.86±0.02 | 2 | This study |
| sbtA-R2 | CCACCAATCAAAAAGGCCAG (20) | *sbtA* | High-affinity bicarbonate/sodium symporter | MAE_62090 | AP009552.1 |  |  | 2 | This study |
| chpX-F | CCTGTCAAGTCCTCCTCTCAT (21) | *chpX* | Low-affinity CO_2_ uptake system | IPF_1842 | AM778957.1 | 113 | 1.90±0.02 | 1 | Sandrini *et al.*, 2015a |
| chpX-R | TTCAGGATACCCACTACCTCG (21) |  |  |  |  |  |  | 1 | Sandrini *et al.*, 2015a |
| chpY-F | ATATCGCCAAAATGCCGACC (20) | *chpY* | High-affinity CO_2_ uptake system | IPF_1545 | AM778958.1 | 114 | 1.80±0.01 | 1 | Sandrini *et al.*, 2014 |
| chpY-R | GACATCATCCGCACCTGTTC (20) |  |  |  |  |  |  | 1 | Sandrini *et al.*, 2014 |
| ccmR-F2 | CCTACCGTCTCAACCCAAGT (20) | *ccmR* | Transcriptional regulator of CCM genes | IPF_1549 | AM778958.1 | 109 | 1.88±0.01 | 1 | Sandrini *et al.*, 2014 |
| ccmR-R | ACAGTAATTCCTGACCCGCTT (21) |  |  |  |  |  |  | 1 | Sandrini *et al.*, 2014 |
| ccmR2-F | TCCTTGGGATAAACCACATACCA (23) | *ccmR2* | Transcriptional regulator of CCM genes | IPF_2166 | AM778949.1 | 204 | 1.88±0.02 | 1 | Sandrini *et al.*, 2014 |
| ccmR2-R | TTTTCTCGACCATGGCATCAC (21) |  |  |  |  |  |  | 1 | Sandrini *et al.*, 2014 |
| rbcX-F | CGGATCATGACGGTAAGAGAACA (23) | *rbcX* | RuBisCO chaperone, in same operon as genes of small and large subunit of RuBisCO | IPF_2531 | AM778933.1 | 157 | 1.87±0.01 | 1,2 | Sandrini *et al.*, 2015a |
| rbcX-R | ATTCCGATGTCTCTGGTTGACT (22) |  |  |  |  |  |  | 1,2 | Sandrini *et al.*, 2015a |
| ccmM-F | AAGTCCACACCTTCTCTAACCTC (23) | *ccmM* | Carboxysomal protein; in same operon as other carboxysomal genes | IPF_5695 | AM778933.1 | 118 | 1.88±0.01 | 1 | Sandrini *et al.*, 2014 |
| ccmM-R | CTGTCGTCGCCAATGTGAA (19) |  |  |  |  |  |  | 1 | Sandrini *et al.*, 2014 |
| ccaA1-F | ACTCCTGCGGTTAATACTGTGG (22) | *ccaA* | Carboxysomal carbonic anhydrase | IPF_5538 | AM778919.1 | 97 | 1.89±0.02 | 1 | Sandrini *et al.*, 2014 |
| ccaA1-R | GATAAATGCGATCAGCTTGGGAG (23) |  |  |  |  |  |  | 1 | Sandrini *et al.*, 2014 |
| mcyB-F | ATCCCATGCTCAGAGACGTT (20) | *mcyB* | Microcystin synthesis | IPF_375 | AM778952.1 | 163 | 1.87±0.02 | 1,2 | Sandrini *et al.*, 2014 |
| mcyB-R | AGATGTCCGCAGGGATTCAT (20) |  |  |  |  |  |  | 1,2 | Sandrini *et al.*, 2014 |
| gvpC-F | GTAATTGAGGACAACCCCATGC (22) | *gvpC* | Structural component of gas vesicles | MAE_37620 | AP009552.1 | 153 | 1.89±0.02 | 1 | This study |
| gvpC-R | TGCCTGTTCTTGCGCTTG (18) |  |  |  |  |  |  | 1 | This study |
| isiA-F | CTTTAGGCTTTGGAGTCGGAGA (22) | *isiA* | Chlorophyll-binding protein | IPF_5322 | AM778872.1 | 117 | 1.89±0.01 | 1 | This study |
| isiA-R | GGTGAAATAAGGCTCCTGCTC (21) |  |  |  |  |  |  | 1 | This study |
| flv4-F | GATCCCCACGAAGTCAGAGA (20) | *flv4* | Photoprotection of PSII | IPF_2586 | AM778929.1 | 166 | 1.89±0.01 | 1 | This study |
| flv4-R | GTTCATCTTCCCCACCTCCT (20) |  |  |  |  |  |  | 1 | This study |

Locus tags are based on the genomes of *Microcystis* strain PCC 7806 (IPF) and NIES-843 (MAE).

Amplification efficiencies of the different primer pairs are based on *n* = 27-86 amplification curves.

**References**

Sandrini G., Cunsolo, S., Schuurmans, J.M., Matthijs, H.C.P. and Huisman, J. (2015a) Changes in gene expression, cell physiology and toxicity of the harmful cyanobacterium *Microcystis aeruginosa* at elevated CO_2_. *Front Microbiol* **6:** 401.

Sandrini. G., Jakupovic, D., Matthijs, H.C.P., and Huisman, J. (2015b) Strains of the harmful cyanobacterium *Microcystis aeruginosa* differ in gene expression and activity of inorganic carbon uptake systems at elevated CO_2_ levels. *Appl Environ Microbiol* **81:** 7730–7739.

Sandrini, G., Matthijs, H. C. P., Verspagen, J. M. H., Muyzer, G., and Huisman, J. (2014) Genetic diversity of inorganic carbon uptake systems causes variation in CO_2_ response of the cyanobacterium *Microcystis*. *ISME J* **8:** 589–600.
